# Pharmacogenetic Considerations in Caffeine Toxicity: Insights Prompted by the “Death Coffee” Case Series

**DOI:** 10.1002/npr2.70027

**Published:** 2025-06-05

**Authors:** Yuji Kamikubo

**Affiliations:** ^1^ Department of Pharmacology Juntendo University Faculty of Medicine Bunkyo‐ku Tokyo Japan

**Keywords:** caffeine, CYP1A2, genetic polymorphisms

I read the case series by Bordbari et al. (2025) describing severe clinical complications following the consumption of “Death Coffee” in Iran with great interest [1]. The report compellingly highlights the emerging public health risks associated with new, high‐caffeine beverages, documenting alarming toxicity, including hallucinations, from just a single cup in several individuals. This work serves as a crucial alert to clinicians and public health authorities.

While the authors provide valuable clinical details for each of the five cases, I believe further discussion regarding potential contributing factors, particularly inter‐individual variability in caffeine metabolism influenced by genetic background, could provide valuable additional context. The report notes the patients were located in Iran, but specific details regarding ethnicity or genetic predispositions, which can significantly impact drug and xenobiotic metabolism, were understandably not the focus of the case series format.

It is well‐established that caffeine metabolism exhibits substantial variation among individuals, primarily due to genetic polymorphisms in the cytochrome P450 1A2 (CYP1A2) gene, the primary enzyme responsible for caffeine clearance. Key polymorphisms, such as *CYP1A2* ‐163C > A (rs762551), categorize individuals into “fast,” “intermediate,” or “slow” metabolizer phenotypes, leading to marked differences in caffeine half‐life and exposure following a standard dose [2, 3].

Furthermore, the allele frequencies of these functional *CYP1A2* polymorphisms vary considerably across different ethnic and geographic populations [4]. However, understanding the specific genetic landscape within the Iranian population is complex. While the prevalence of specific “slow metabolizer” genotypes requires careful consideration, a recent review highlights a high frequency (approx. 60% allele frequency) of the CYP1A21F allele (rs762551), associated with fast/inducible metabolism, within this population [5].

While individuals carrying slow metabolizer alleles (e.g., CYP1A21C) would be expected to have increased susceptibility to toxicity due to higher plasma concentrations, the high prevalence of the fast/inducible 1F allele in Iranians complicates this interpretation. Several factors might explain the severe toxicity reported by Bordbari et al. despite the population's tendency toward fast metabolism. First, the caffeine dose in “Death Coffee” may have been extraordinarily high, potentially overwhelming even fast metabolic pathways due to saturable kinetics. Second, the affected individuals might belong to the subset of the population carrying less common slow‐metabolizing alleles or genotypes, or possess other genetic factors influencing sensitivity, such as ADOR A2A polymorphisms [4]. Third, environmental factors significantly modulate CYP1A2 activity; for instance, smoking is a known inducer, particularly for the *1F* allele, while other medications or dietary factors could act as inhibitors, although such details were not available in the case series. Therefore, a simple genotype–phenotype correlation is challenging without individual data.

Therefore, while the possibility remains that the individuals experiencing severe reactions possessed specific genetic predispositions (e.g., slow metabolizer genotypes or high sensitivity profiles), the observed toxicity could also result from the extreme caffeine dose itself, potentially modified by unmeasured environmental or genetic factors. While genotyping was likely not feasible in the acute setting described, considering the potential prevalence and complex interplay of different CYP1A2 metabolizer phenotypes and modifying factors within the Iranian population could be informative for risk assessment and public health messaging regarding high‐caffeine products in the region.

In conclusion, the important case series by [1] underscores the dangers of unregulated high‐caffeine products. Integrating the perspective of pharmacogenetic variability in caffeine metabolism, supported by literature evidence [6, 7, 8], may further elucidate the differential susceptibility to caffeine intoxication. This understanding highlights the potential need for clearer labeling of caffeine content, regulatory oversight of such ultra‐high caffeine products, and potentially targeted public health advice for populations or individuals potentially at higher risk. Considering both genetic predispositions (such as CYP1A2 metabolizer status) and relevant nongenetic factors could help tailor preventative strategies in diverse populations. Thus, incorporating pharmacogenetic insights into public health strategies and risk assessments for high‐caffeine products is increasingly vital, particularly when considering diverse populations and the growing market for such beverages.

## Ethics Statement

The author has nothing to report.

## Consent

The author has nothing to report.

## Conflicts of Interest

The author declares no conflicts of interest.

## Data Availability

Data sharing not applicable to this article as no datasets were generated or analysed during the current study.
